# In Reply: Letter to the Editor on “A Case Report of Delayed, Severe, Paroxysmal Muscle Cramping after Chilean Rose Tarantula (*Grammostola rosea*) Envenomation”

**DOI:** 10.5811/cpcem.48543

**Published:** 2026-03-07

**Authors:** Jon B Cole, Brian T Gooley, Kirk A Hughes, Mark P Gooley, Daniel E Keyler, Richard S Vetter

**Affiliations:** * Minnesota Poison Control System, Minneapolis, Minnesota; † Mayo Clinic, Rochester, Minnesota; ‡ University of Minnesota, Minneapolis, Minnesota; § University of California Riverside, Riverside, California

To the Editor:

We appreciate the author’s interest in our case report. We agree with his assertion that species identification is important in spider envenomation cases. Indeed, we sought to follow best-practice guidelines in reporting our case, as outlined in the article by Kong and Hart. Regarding the spider in our case, we took the following steps to identify the spider.

1. First, the spider was identified a priori by both the patient and the spider’s owner as *Grammostola rosea*.
2. Second, we had additional images to help us identify the spider, not provided for conciseness of the manuscript.
3. Third, one of our co-authors, KAH, who has over 25 years of experience as a certified specialist in poison information as well as personal experience with tarantulas, agreed with the identification of the spider in the photos.
4. To ensure the identification of the spider was correct, and to ensure proper scientific context for spider envenomations, we recruited two additional authors with decades of experience as recognized international experts in envenomations, DEK and RSV, who also agreed with the species identification based on the photos. Richard Vetter, in particular, is an international expert in spider envenomation.

While we feel reasonably confident in our identification, given the feedback from this letter-writer, we sent the photo in the article to three additional experts in arachnology, who had differing opinions, and noted the spider could be *Grammostola rosea* as we wrote, but also *Grammostola albopilosus, Tliltocatl albopilosus*, or a *Lasiodora* species.

Given that there is some uncertainty with the identification of the spider, we agree that a limitation of this case report is that the tarantula could have been misidentified. The spider was identified by its keeper and several experts as *Grammostola rosea*, but also has features of *Grammostola albopilosus, Tliltocatl albopilosus*, and *Lasiodora* species. Nevertheless, emergency clinicians should remain aware of the possibility of delayed severe paroxysmal muscle cramping with tarantula envenomation from any species.
